# Pandemic response management framework based on efficiency of COVID-19 control and treatment

**DOI:** 10.2217/fvl-2020-0368

**Published:** 2020-12-16

**Authors:** Mustapha D Ibrahim, Fatima AS Binofai, Reem MM Alshamsi

**Affiliations:** ^1^Industrial Engineering Technology, Higher Colleges of Technology, PO Box 7947, Sharjah, United Arab Emirates

**Keywords:** COVID-19, data envelopment analysis, efficiency, pandemic control, pandemic response framework

## Abstract

**Aims:** The existing response management system for pandemic disease fell short of controlling COVID-19. This study evaluates the response management relative efficiency of 58 countries in two stages, using two models. **Materials & methods:** Data envelopment analysis was applied for efficiency analysis. **Results:** 89.6% of countries were inefficient in pandemic control and 79% were inefficient in treatment measures. Sensitivity analysis underlines resources as a critical factor. Further examination points to absence of a robust and uniform mitigation measure against the pandemic in most countries. **Conclusions:** Preventing spread is not only the first line of defense; it is the only line of defense. The lack of a global public health database support system and uniform response compounded inefficiency. A robust pandemic response management framework is developed based on practices of key performers. Action plans are proposed, with a recommendation for a global public health pandemic database monitoring and support system as the nucleus.

## Graphical abstract

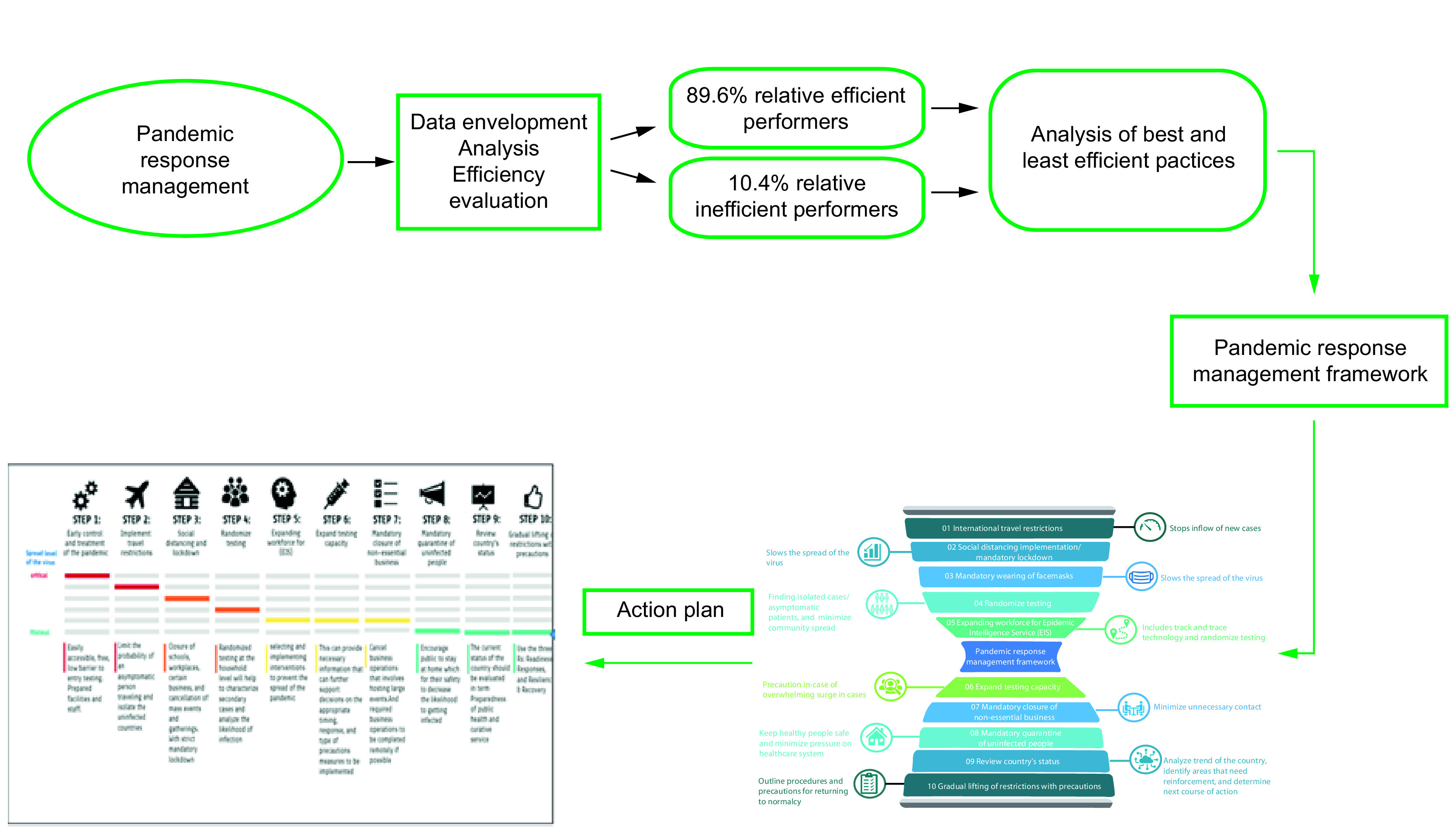

## Background

In a pandemic, gaps develop between the existing protocols, resource availability, needs and infrastructures. It is of prime importance that systems analyze their pandemic response plans to understand their preparedness and response and, more importantly, their ability to adopt modifications based on information about the pandemic. The goals of a successful pandemic management system are to continuously assess needs, identify resources, plan the response and implement the plan [1]. SARS-CoV-2, the coronavirus responsible for COVID-19, is a labeled a highly efficient transmitted virus due to its person-to-person transmission even by individuals without apparent symptoms [2]. The UN Secretary-General called for *“immediate health responses required to suppress transmission of the virus, to end the pandemic and to tackle the many social and economic dimensions of this crisis*" [3].

Pandemic response management is essential for decreasing the spread of a virus and its associated morbidity and mortality. Establishing new protocols and estimating the demand for resources such as physicians, hospital beds, personal protective equipment (PPE), ventilators, emergency transport vehicles and nurses can help guide decision-makers’ control of the virus. Different countries have adopted different measures toward COVID-19 but, so far, previously established standards of pandemic control have fallen short of effectively controlling the disease. Given the fluidity of the pandemic, a response framework based on efficiency analysis of contagion control and treatment can give great insights on what to do and when to do it. An important lesson so far is that the timing and sequence of response measures are imperative in pandemic response. It is important to analyze countries that have performed relatively well in managing the virus considering the multiple factors involved. This study aims to evaluate the efficiency of COVID-19 response and treatment, to learn from the best and worst performing countries and to propose a robust framework that adapts to more severe pandemics like COVID-19. Results of performance analysis in phases and stages of the virus, with inference on practical measures in a framework based on successes and failures, will set a new pandemic response standard. Studies such as those of Shirouyehzad *et al*. and Breitenbach *et al*. [4,5] evaluated the technical efficiency of healthcare systems based on COVID-19. However, those studies are void of measures that were taken in stages to combat the surge in the virus spread. In addition, proper representation of the negative outputs of the pandemic were not defined. In this regard, the current study models the negative outputs caused by the pandemic into the efficiency analysis, analyzes different measures by the best and worst performing countries, and proposes a robust pandemic response framework that can withstand severe pandemics like COVID-19.

## Global overview of COVID-19

About 200 countries around the world have recorded cases of COVID-19, with around 60 million confirmed cases and 1.4 million deaths globally as of 22 November 2020, according to Johns Hopkins University [6]. The USA, India, Brazil, France and Russia are the top five ranked with severe cases. Countries are trying to control the pandemic by learning from previous experiences of similar pandemics with similar genealogy, such as the SARS outbreak in 2003. However, COVID-19 has a higher transmission rate and has thus resulted in a larger global outbreak [7]. An analysis of International Health Regulations annual report data from 182 countries showed that 57% of countries are capable of preventing, detecting and controlling a novel infectious disease, with the remaining 43% having a lower capacity for preventing and controlling an outbreak [8]. Nonetheless, countries with perceived advanced healthcare systems fell short of controlling COVID-19. Financial and technical supports are required for developing countries whose capacity is insufficient to handle a pandemic [9]. All healthcare resources – physicians, nurses, hospitals, beds and capacity, ventilators, ambulances, PPE and reliable healthcare product supply chains, among others – are in short supply globally. Few countries have been able to balance supply given the rapid rise in demand. Many countries reached maximum capacity in intensive care units. This affects the quality of care rendered to the population, because patients with mild symptoms are turned back to create space for severe cases. As countries are coping with the health, economic, financial, political and educational challenges posed by the pandemic, global relief efforts have increased to support countries that are most hit by the pandemic. The United Arab Emirates donated about 75 tonnes of medical supplies to different nations, including Iran, Italy, Colombia and Kazakhstan, to support these global efforts [10]. China also donated health supplies to Italy, Spain and some Latin American countries [11]. There is a call for international collaboration in research, economic and resource balance to defeat the pandemic.

## Data & methods

### Data description

The process of analyzing COVID-19 pandemic response management is categorized into two stages. Stage 1 considers COVID-19 contagion control efficiency, analyzes countries’ performance in terms of minimizing the spread of the virus and identifies countries that adopted efficient pandemic control measures. Two inputs and one output are considered in this stage. Factors that have been described as critical to the spread are utilized. Population density and COVID-19 confirmed cases are considered as outputs. Population density is the measurement of population per unit area; it is one of the factors known to influence transmissibility of COVID-19, with a moderate risk of infection for people working in areas with high population density [12,13]. The average of 13 International Health Regulations (IHR) core capacity scores is an indicator representing the core capacities that have been achieved by a country at a given point. These 13 indicators have been identified to have connection to COVID-19 and are integral to the preparedness and vulnerability of countries in relation to COVID-19 [14]. They are: legislation and financing, IHR coordination and national focal point functions, zoonotic events and the human–animal health interface, food safety, laboratory, surveillance, human resources, national health emergency framework, health service provision, risk communication, point of entry, chemical events and radiation emergencies. Stage 1 of the analysis is further classified into two phases: stage 1A considers the first 3 months after announcement of the pandemic, and stage 1B examines the subsequent 3 months to see which countries improve or maintain efficient contagion control and the ways in which they achieve these improvements. It is important to note that the number of tests conducted was considered for inclusion; however, a lack of data and the known inconsistencies and unreliability of existing data for most countries led to its exclusion. Nonetheless, the test trends were examined at later stages of the analysis.

Stage 2 of the analysis evaluates the treatment efficiency and management of the pandemic. The number of confirmed COVID-19 cases is an important input in this stage, because it represents the pressure exerted on the healthcare system and constitutes the primary input of the pandemic treatment. Relevant resources, such as number of ventilators, number of testing and amount of PPE were considered for inclusion; however, due to unavailability of data, they were discarded as variables. Other relevant resources with available data (number of physicians per 1000 population and number of hospital beds per 1000 population) were utilized. The percentage of the population with age >65 is an important factor because fatality of the virus is more prevalent in the elderly population and individuals with preexisting health conditions [15,16]. The number of physicians per 1000 population and the number of hospitals per 1000 population are important parameters used to evaluate efficiency of healthcare systems and adequacy of the system capacity [17,18]. This stage uses data from 6 months after the pandemic announcement. Variables for efficiency evaluation are as follows. Stage 1 (COVID-19 contagion control efficiency): inputs are population density [19] and average of 13 IHR core capacity scores [20]; outputs are COVID-19 confirmed cases [20]. Stage 2 (COVID-19 treatment efficiency): inputs are COVID-19 confirmed cases [21], number of physicians per 1000 population [27], number of hospital beds per 1000 population [19] and percentage of population with age >65 years [19]; outputs are COVID-19 related deaths [21] and COVID-19 recovered cases [21].

### Data envelopment analysis

The variables used to model efficiency of COVID-19 control and treatment, present a complex system. Therefore, a robust technique that can handle multiple inputs and outputs in addition to negative outputs (e.g., positive COVID-19 cases and mortality) is required. Data envelopment analysis (DEA) is a performance evaluation technique capable of handling multiple inputs and outputs [22], with abundant empirical applications in healthcare systems and strategies [17,23–25]. DEA has been applied to analyze effects and efficiency of pandemics such as HIV/AIDS [26]. The efficiency of schistosomiasis control programs in Jiangsu Province, China was also analyzed using DEA [27].

DEA is a nonparametric method of efficiency evaluation introduced by Charnes *et al*. [28] under constant return to scale (CRS) to evaluate efficiency of systems known as decision-making units (DMUs). It was later modified by Banker *et al*. [29] with variable return to scale (VRS). Subsequently, various models have been developed, including direction distance function [30] and target setting model [22]. DMUs are generic, taking the form of countries, systems or companies that need evaluation with a set of homogeneous parameters. It constructs a best-practice frontier from the sample observations and measures the radial distance of other observations relative to the frontier [31]. This study utilizes DEA to evaluate the performance of countries in terms of their COVID-19 pandemic management. The DEA efficiency scores show the performance level of each country relative to other countries for the evaluated period. DEA compares the homogeneous units among themselves and accepts the best observation as the efficient frontier, then other observations are benchmarked against that frontier [17].

Efficient pandemic contagion control requires utilization of resources to minimize the spread of the pandemic, in addition to new protocol implementation. Furthermore, efficient pandemic treatment practice with the number of infections and available resources necessitates minimizing the fatality rate and maximizing the number of patients treated. In this context, the DEA model adequately handles such parameters (desirable and undesirable outputs) and objectively evaluates efficiency by accounting for the asymmetry between both types of outputs [32] and alleviating the possibility of biased results due to converting undesirable outputs to their inverse (ratio) [33].

When considering a multiple input and output system [28] the production possibility set (PPS) is defined as:

$$PPS=\left\{\left(X,Y\right)|\sum_{j=1}^{n}X_{j}\lambda_{j}\leq X,\sum_{j=1}^{n}Y_{j}\lambda_{j}\geq Y,\sum_{j=1}^{n}\lambda_{J}=1,\lambda_{j}\geq 0,\;j=1\ldots n\right\},$$

where $X_{j}=(x_{1j},\cdots ,x_{mj})\;and\;Y_{j}=(y_{1j},\cdots ,y_{sj})$ represents the observed *m*-inputs and *s*-outputs of j=1,…,n DMUs. Chambers introduced a directional distance efficiency measure by projecting units $\left(x_{0},y_{0}\right)$ to a preassigned coordinate $g=(-g_{x}^{-},g_{y}^{+})\neq 0_{m+s},g_{x}^{-}\in {ℜ}^{m}\;and\;g_{y}^{+}\in {ℜ}^{s}$ in a direction *β* [34]. Equation 1 illustrates the linear program associated to the estimation.

(Eq. 1) $$\begin{array}{l} \underset{\beta ,\;\lambda }{Max}\;\;\;\;\beta \\ Subject\;to\\ X\lambda \leq x_{0}-\beta g_{x}^{-}\\ Y\lambda \geq y_{0}+\beta g_{x}^{+}\\ \lambda \geq 0 \end{array}$$

The optimal solution of Equation 1 corresponds to the CRS efficiency $\beta^{*}{}_{CRS}$. If $\beta_{CRS}^{*}=0$, the unit under evaluation is technically efficient, whereas $\beta_{CRS}^{*}>0$ signifies an inefficient unit. Correspondingly, the VRS model is achieved by adding $\sum_{j=1}^{n}\lambda_{j}=1$ , as shown in Equation 2. The optimal solution of Equation 2 is VRS efficient if $\beta_{VRS}^{*}=0$ and inefficient if $\beta_{VRS}^{*}>0$. Consequently, the scale efficiency from the directional model is achieved as follows: $SE=\beta_{CRS}^{*}-\beta_{VRS}^{*}$

(Eq. 2) $$\begin{array}{l} \underset{\beta ,\;\lambda }{Max}\;\;\;\;\beta \\ Subject\;to\\ X\lambda \leq x_{0}-\beta g_{x}^{-}\\ \begin{array}{l} Y\lambda \geq y_{0}+\beta g_{x}^{+}\\ \sum_{j=1}^{n}\lambda_{j}=1 \end{array}\\ \lambda \geq 0 \end{array}$$

Along with positive output of a system, undesirable outputs are sometimes observed, such as hazardous waste in an environmental context or mortality/fatality in healthcare. Most efficiency evaluation models do not account for the asymmetry between both types of outputs, which leads to erroneous efficiency estimation. Incorporation of the characteristics of undesirable outputs into DEA efficiency estimation relies on a directional measure that handles desirable and undesirable outputs differently [32].

The PPS is redefined as follows: the initial output vector of $i=1,2,\ldots ,s.y\in {ℜ}_{++}^{s}$ is divided into desirable and undesirable $y=\left(y^{d},y^{u}\right)$, with $y^{d}\in {ℜ}_{++}^{q}$ respectively. This is expressed into the following reference $PPS_{CRS}=\left\lfloor (x,\;y^{d},\;y^{u})|x\geq x\lambda ,\;y^{d}\leq y\lambda ,\;y^{u}=y\lambda ,\;y\geq 0\right\rfloor$, designating undesirable outputs as weakly disposable [35]. To prevent the inconsistencies in the method of [30], the method of [36] is used to define directional efficiency, resulting in an increase in desirable outputs and a decrease in undesirable outputs from the same inputs. Therefore the directional efficiency measure corresponds to the solution of Equation 3.

(Eq. 3) $$\begin{array}{l} \underset{\beta ,\;\lambda }{Max}\;\;\;\;\beta \\ Subject\;to\\ X\lambda \leq x_{0}\\ \begin{array}{l} Y^{d}\lambda \geq y_{0}^{d}+\beta y_{0}^{d}\\ Y^{u}\lambda \leq y_{0}^{u}-\beta y_{0}^{u}\\ Max\{y_{i}^{u}\}\geq y_{0}^{u}-\beta y_{0}^{u_{0}} \end{array}\\ \lambda \geq 0. \end{array}$$

The optimal solution of Equation 3 is $\beta_{CRS}^{*}$, if $\beta_{CRS}^{*}=0,\;with\;\lambda =1,\;\lambda_{j}=0(j\neq 0)$, the unit under evaluation is directionally efficient. Otherwise, $\beta_{CRS}^{*}>0$ signifies an inefficient unit.

**Figure 1.**
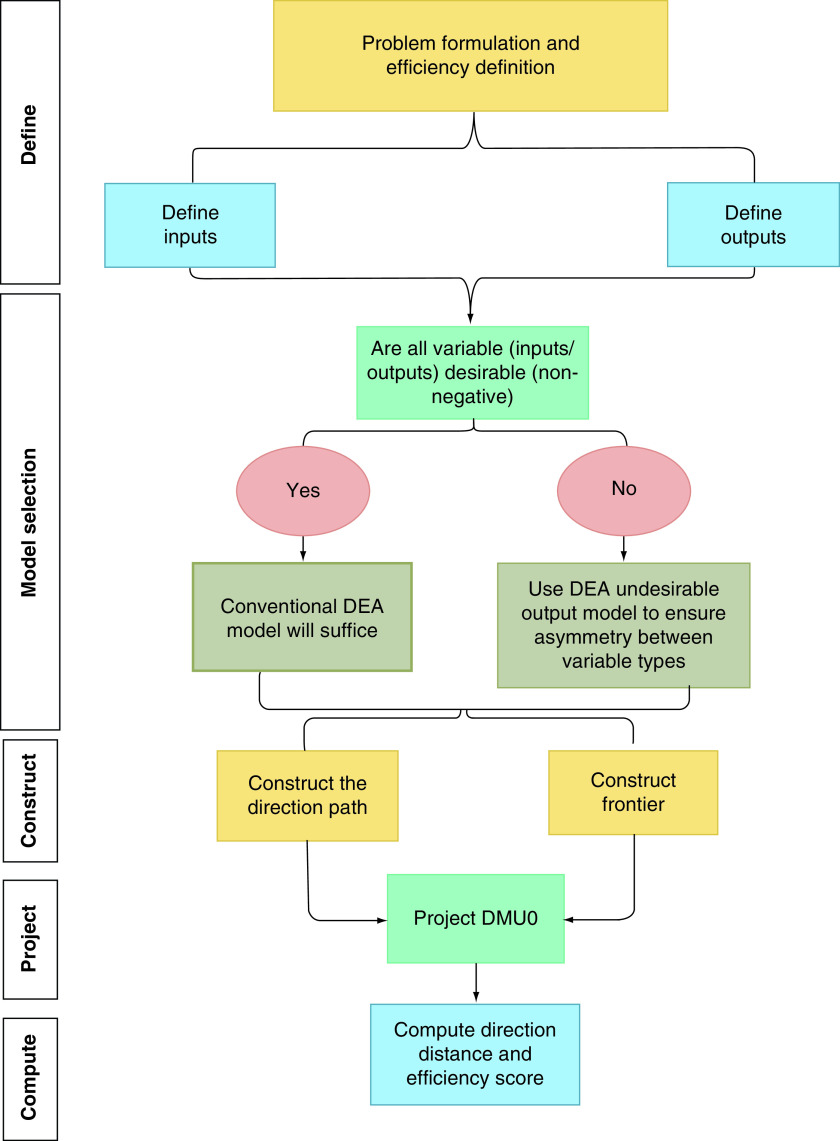
Efficiency evaluation flow diagram.

Given the different frontier estimating methods, this application can be difficult to understand for non-experts on frontier based models. Figure 1 presents a flow diagram illustrating the development and implementation of the model.

## Results

The COVID-19 data of 15 July 2020 were the latest data extracted. Across the period, confirmed cases increased in all measures. The USA recorded the highest number of confirmed cases in both the first 3 months and the subsequent 3 months. There was an increase in the average confirmed cases among the countries considered. China recorded the minimum confirmed cases, with a 97% decrease compared with the preceding 3 months (see Supplementary Table 1: descriptive statistics).

**Figure 2.**
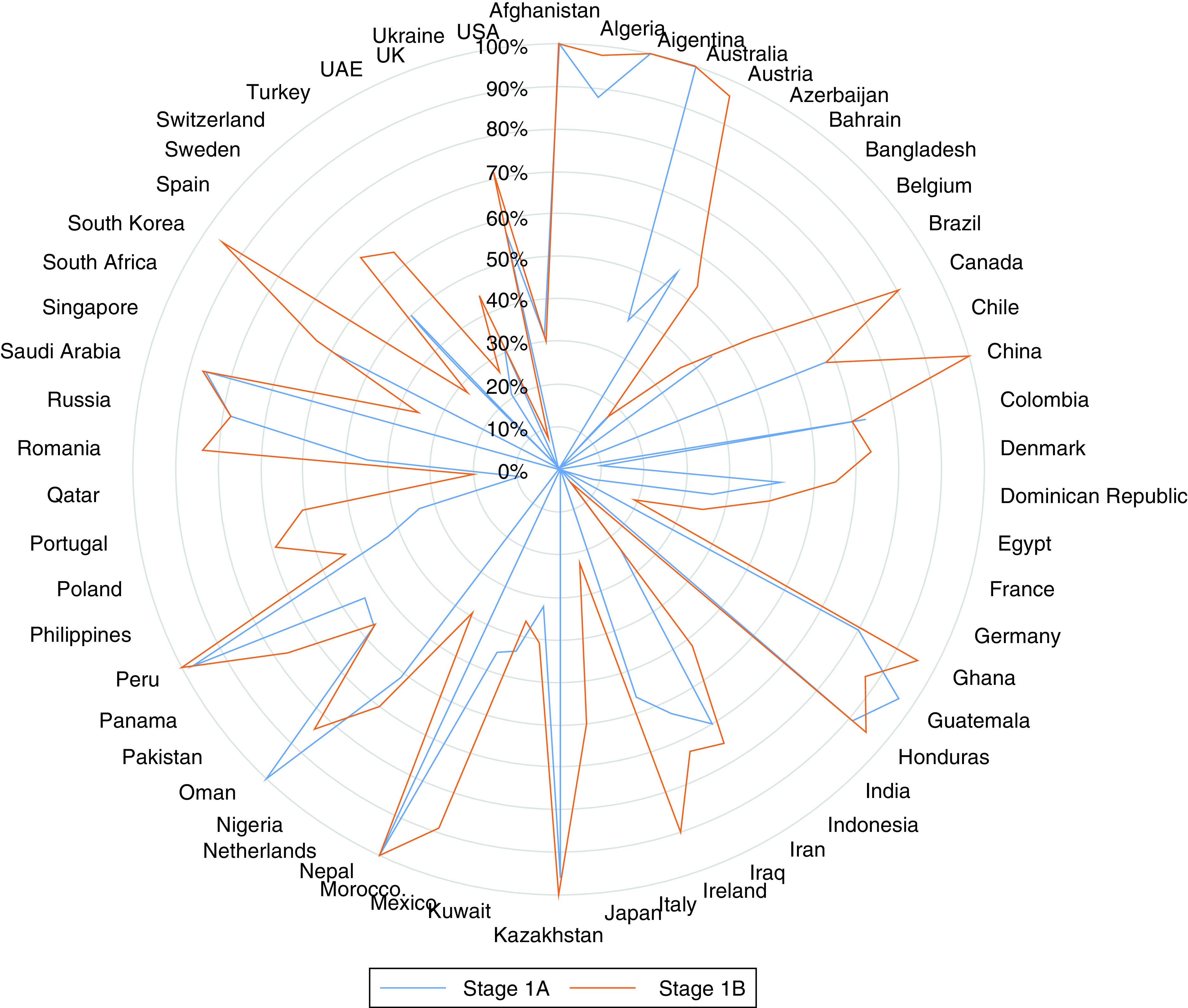
Efficiency of contagion control.

To ensure a balanced dataset, 58 countries were considered in stage 1 and 57 countries in stage 2. Figure 2 presents the efficiency scores for contagion control of stage 1A and stage 1B. 89.6% of the countries evaluated were inefficient, with an average efficiency of 45.6% in stage 1A. The average contagion control efficiency improved to 64.3% with about 87.9% of the countries still inefficient in stage 1. China and South Korea showed a remarkable improvement, with 99.7 and 95.2% efficiency improvement respectively, in the second phase of contagion control (Figure 2). Other significant improvements included Denmark (64%), Switzerland (63.1%), Austria (59%), Japan (57.8%), Bahrain (52.3%), Portugal (50.7%) and Morocco (54.9%). Australia, Argentina, Afghanistan, Kazakhstan and Peru were consistently efficient. Countries such as Oman, Guatemala, Mexico, Columbia and Bangladesh performed worse in stage 1B, with negative efficiency improvements of 15.3, 9.2, 7.7, 3.3 and 2%, respectively. Pakistan, the USA, Brazil and Chile showed no improvement in the second phase despite their significant inefficiency in the first phase. Supplementary Table 2 illustrates the numerical contagion efficiency scores.

**Figure 3.**
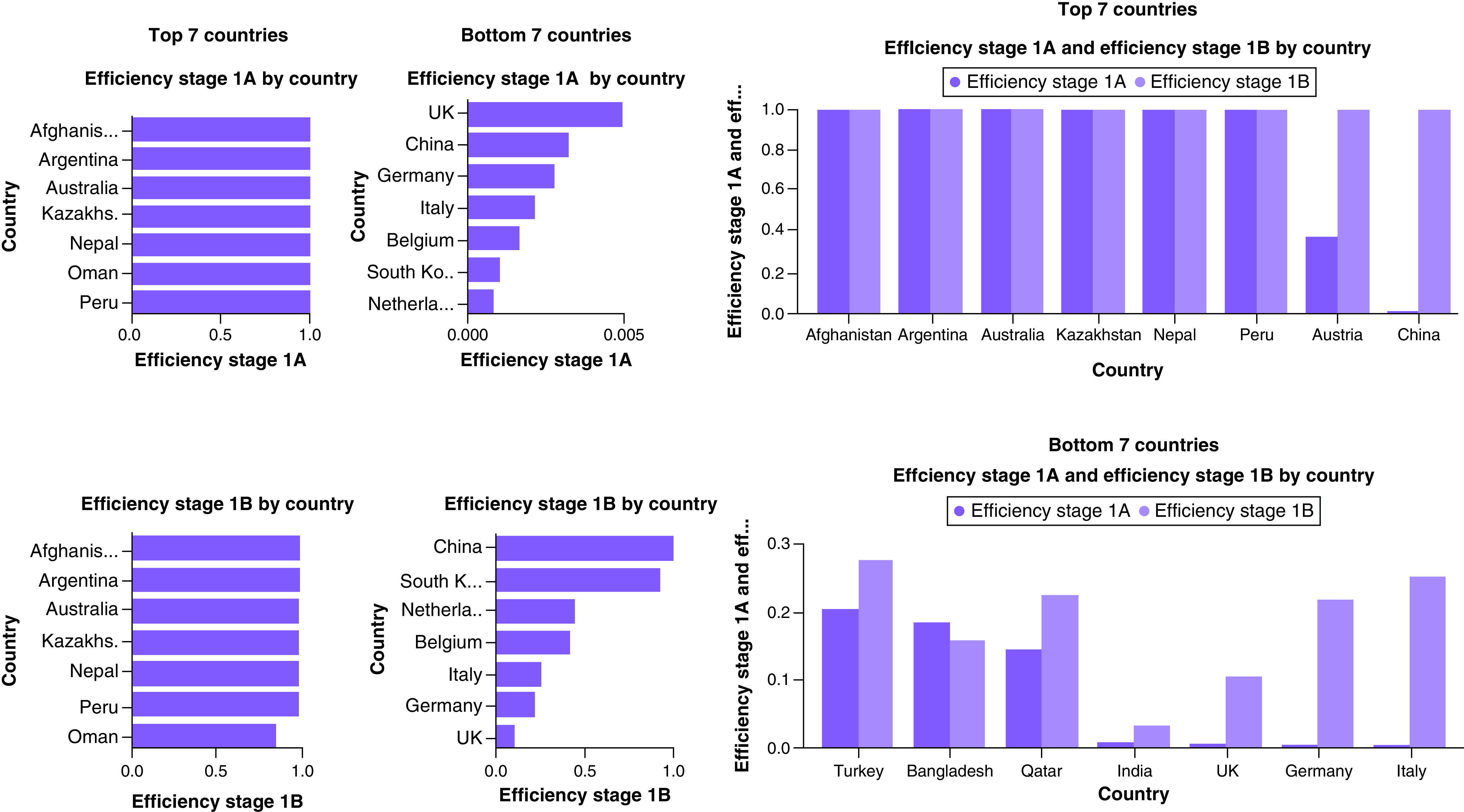
Summary of the seven most and least efficient countries.

Figure 3 presents a summary analysis of contagion control efficiency. Changes in efficiency of the most and least efficient countries are illustrated in Supplementary Figure 1.

**Figure 4.**
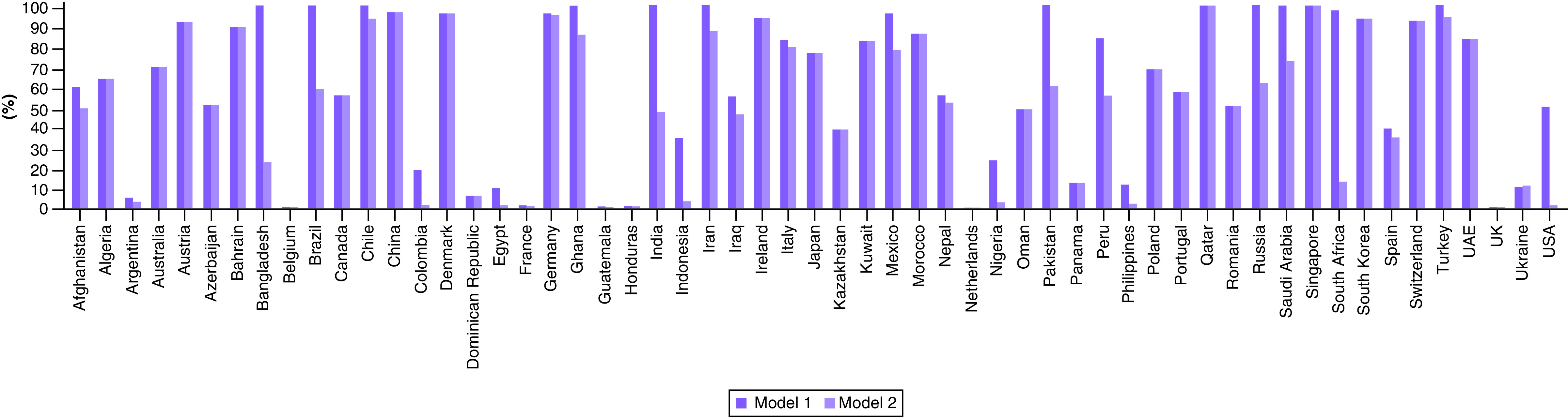
Efficiency of COVID-19 treatment (models 1 & 2).

The second stage of the analysis looks at countries’ efforts toward treating the virus during the evaluated period. Consideration of the efficacy and efficiency of the drugs used for treatment is beyond the scope of this study, which focuses on identifying the countries that have done a relatively good job of minimizing COVID-19-related deaths and maximizing recovered cases. Figure 4 presents the results of COVID-19 treatment (model 1) and sensitivity analysis using only COVID-19 confirmed cases as input (model 2). Model 1 indicates that 79% of the countries considered were inefficient in treating the virus, with an average efficiency score of 62.1%. A robustness check of the result, performed using sensitivity analysis by considering only confirmed cases as inputs, shows 96.5% of the countries were inefficient in treating the virus, with an average efficiency score of 51%. Supplementary Table 3 illustrates the numerical treatment efficiency scores.

## Discussion

The efficiency analysis of control and treatment of COVID-19 across 58 countries for the first 6 months of the pandemic provides insight on response management performance of different countries. Countries like Austria, Bahrain, China, Denmark, Germany, Ghana, Ireland, Italy, Morocco, Qatar, Singapore, Switzerland, Turkey and the United Arab Emirates showed a consistent efficiency in both treatment efficiency analysis models. The United Kingdom, Netherlands, Belgium, France, Guatemala and Honduras (among others) were consistently inefficient in both treatment efficiency analysis models. The USA, Brazil, Russia, Pakistan, Bangladesh, India and South Africa showed above 35% decrease in efficiency in the second model.

Countries that have zero or negative changes in efficiency of contagion control between stage 1A and stage 1B exhibit an inefficient COVID-19 treatment. Therefore, for most countries, it is important to note that preventing the spread of the virus is not only the first line of defense; it is the only line of defense. In addition, sensitivity analysis highlights the significance of resources such as number of physicians and hospitals as critical factors toward defeating the pandemic. This is supported by the significant drop in efficiency in countries such as Bangladesh, Brazil, India, Nigeria, Pakistan and South Africa.

### Pandemic response management framework & action plan

**Figure 5.**
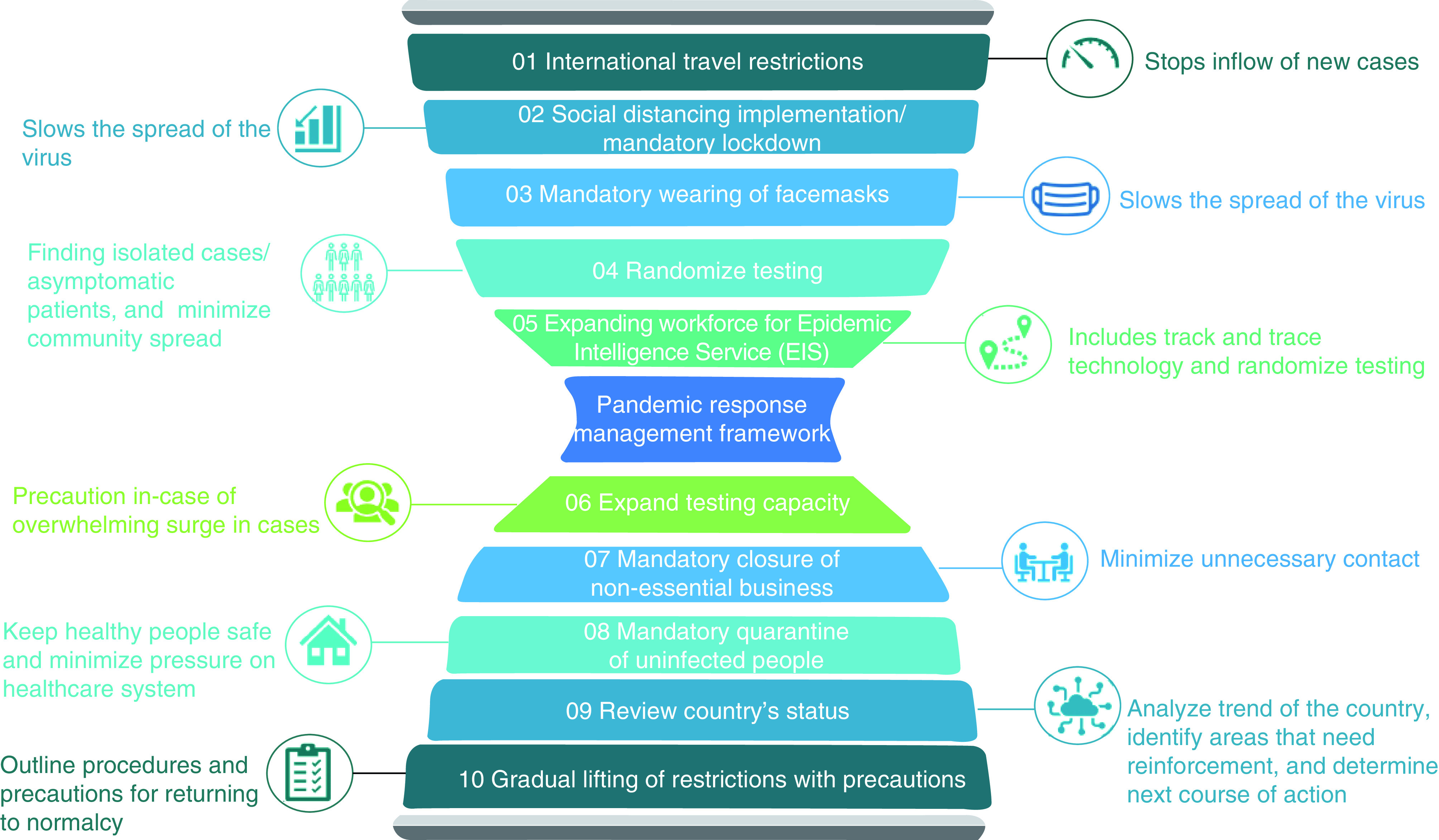
Pandemic response management framework.

The gross inefficiency of COVID-19 contagion control across the 58 countries evaluated in this study is indicative of the absence of a robust pandemic response management framework capable of controlling a pandemic of such magnitude. Practices of the best and worst performing countries are examined to propose a robust pandemic response management framework. Countries with high stage 1A scores and a significant positive difference in efficiency scores are analyzed (Table 1). Countries with negative and zero difference in stage 1 efficiency scores are also examined (Table 2). Actions of these key countries were used to develop the pandemic response management framework illustrated in Figure 5.

**Table 1.** Countries with high stage 1A and significant positive difference in efficiency.

Country	Practices in stage 1 phase 1	Practices in stage 1 phase 2	First case reported^†^	Testing strategies	Ref.
Austria	Closure of schools, restaurants and most businesses Social distancing International travel restriction Mandatory wearing of face masks	Similar strategy is maintained	25 February; 2 cases	Testing of the following groups should be prioritized (in decreasing order of importance): • Testing of hospitalized patients with SARI • Testing all people with ARI in long-term care facilities (or, as a minimum, the first cases to confirm an outbreak in closed settings) • Elderly people and those with underlying chronic medical conditions (e.g., lung disease, cancer, heart failure, cerebrovascular disease, renal disease, liver disease, hypertension, diabetes and immunocompromising conditions) who show signs of acute respiratory illness because they are in need of immediate support more than other groups • Testing of subsets of patients with ARI or ILI in sentinel outpatient settings	[37–39]
Australia	School closure Social distancing International travel restriction Home quarantine	Tracing app Social distancing Managing the demand on health resources Increased health system capacity Mandatory quarantine of 14 days for travelers Isolation and quarantine for contacts of confirmed cases Continuation of border surveillance Travel restrictions	25 January; 3 cases	COVID-19 test prioritization in certain settings and institutions: • Aged care facilities • Residential care facilities • Correctional facilities • Other institutions • Remote Aboriginal communities Test sample prioritization category includes: • ICU inpatients • ED inpatients • Ward inpatients • Healthcare inpatients • Public health outbreak control workers • Essential services workers	[38,40,41]
Bahrain	Patient isolation and treatment International travel restriction Closure of schools, universities and some businesses Social distancing Contact tracing app	Social distancing Random COVID-19 testing for citizens and residents. Mandatory wearing of face masks	24 February; 2 cases	No available information	[38,42]
China	Mass screening of school-aged children for febrile illness Closure of schools, workplaces, roads and transit systems Workplace distancing Cancellation of public gatherings Mandatory quarantine of uninfected people without known exposure to SARS-CoV-2 Social distancing Isolation and quarantine of patients with SARS and their contacts Lockdown	International travel restriction Social distancing Establishing the Joint Prevention and Control Mechanism	11 January; 41 cases	Mandatory nucleic acid testing for the virus should cover the key groups, including: • Close contacts of confirmed COVID-19 patients • Travelers • Patients at fever clinics • Patients to be hospitalized • Healthcare staff	[38,45–47]
Denmark	Closure of schools, universities, entertainment industries and other services Social distancing Closure of borders Home quarantine	Similar strategy maintained	27 February; 1 case		[38–40]
Italy	Complete lockdown Strict self-isolation measures	Increase in healthcare system capacity Training of healthcare employees Sufficient supply of medical supplies and PPE Movement tracing Social media campaigns Closure of all nonessential activities	29 January; 2 cases		[38,39,43,44]
Japan	Partial state of emergency Wearing of face masks School closure Three-pillar plan: • Identification of early infected clusters • Selective PCR testing • Voluntary stay-home orders Social distancing	Nationwide emergency Wearing face masks Closure of some business	14 January; 1 case	No information	[38,47]
Portugal	Early imposition of lockdown Home quarantine Restrictions on social and religious gatherings School closure	Mandatory wearing of face masks Reopening of some services with new rules Maintenance of restrictions on religious gatherings	2 March; 2 cases		[38,39,48,49]
South Korea	Isolation and quarantine for people who contacted with confirmed cases Transformation of public facilities and retreat centers owned by private corporations into temporary isolation wards to prevent transmission within households Expansion of testing capacity Expansion of the EIS workforce Social distancing	Self-diagnosis app Mandatory quarantine of 14 days for travelers Operating triage rooms (expanding tests)	19 January; 1 case	No information	[38,50]
Switzerland	Closure of borders Mandatory quarantine of 14 days for travelers Restrictions in religious, entertainment and personal services centers Gradual easing of restrictions in personal services and family funerals	Contact tracing app for people who contacted infected cases Mandatory wearing of face masks in public transportation and when the distance is <1.5 m in public places Reopening the borders Gradual easing of restrictions in other services	24 February; 1 case	No information	[38,51]

† All dates in 2020.

ARI: Acute respiratory illness; ED: Emergency department; EIS: Epidemic Intelligence Service; ICU: Intensive care unit; ILI: Influenza-like illness; PPE: Personal protective equipment; SARI: Severe acute respiratory illness.

**Table 2.** Countries with negative and zero efficiency difference in stage 1.

Countries	Practices in stage 1A	Practices in stage 1B	Ref.
Bangladesh	Low number of COVID-19 tests conducted Mandatory lockdown Reduced international flights Imposed thermal scanner checking	Mandatory lockdown Severe shortage of testing kits Lack of awareness from the general public Lack of information on COVID-19 confirmed cases Low availability of healthcare workforce	[52–54]
Brazil	Low number of COVID-19 tests conducted (3462 per 1 million people) Urban communities that hinder early implementation of social distancing	Lack of public awareness (30% of Brazilians aged 15–64 years are illiterate)	[55]
India	International travel restrictions Mandatory lockdown	Intensive campaign and guidelines for personal hygiene, surveillance, contact tracing, quarantine, diagnosis, laboratory tests and management	[56]
Mexico	Late response to the pandemic by suspending all nonessential activities, though with few details on its implementation and enforcement Lack of PPE	Minimal testing Use of poor-quality PPE increased the risk of transmission High percentage of infected healthcare personnel Lack of safety provisions in healthcare systems	[57]
Sweden	Absence of mandatory mask wearing Absence of social distancing implementation Absence of mandatory closure of nonessential businesses Quarantine implemented only if people showed symptoms (underestimating the risk of asymptomatic people transmitting the virus)	Absence of mandatory mask wearing Absence of social distancing implementation Absence of mandatory closure of nonessential businesses International travel restrictions introduced at a late stage; passengers were not screened or quarantined	[58]
Oman	International travel restrictions Social distancing Home quarantine Scaling of diagnostic tests and medical resources	Mandatory lockdown as cases continued to rise Limited availability of diagnostic tests initially, delays in diagnosis, limited access to medical treatment in areas highly populated with labor workers	[59]
United Kingdom	PPE shortage Minimal testing Track and trace initially implemented but later disregarded Late implementation of mandatory lockdown 18% of physicians absent due to COVID-19 infection or quarantining Delayed social distancing	Expansion of testing capacity Implementation Ranked as third lowest in number of hospital beds per 1000 population among 20 countries Late closure of schools, business and other social activities	[60,61]
USA	Delayed response due to test kit shortages Fewer COVID-19 tests per capita (compared with South Korea, which announced the first cases on the same day) Absence of clear co-ordination/uniformity of protocols. No surveillance testing program to screen for COVID-19 spread in asymptomatic people.	PPE shortage Absence of clear co-ordination/uniformity of protocols.	[56,61]

PPE: Personal protective equipment.

Clear, uniform and regular public communication has proved effective in informing the population on the severity and importance of adhering to new protocols. Furthermore, upscaling vigilance coupled with the proposed pandemic response management framework could be more effective.

The mandatory lockdowns that have been imposed are not a sustainable approach, due to their economic and health effects. Step 10 of the framework suggests gradual lifting of restrictions with precautions. The following can be incorporated as restrictions on traveling and other aspects of human life are lifted:

- The use of infrared thermal imaging scanning;
- The use of QR codes for all international travelers entering a country; the traveler will be asked to scan a QR code that takes them to an online declaration form containing contact information and determining whether they have COVID-19 symptoms. In addition, it can be used in hospitals to track confirmed cases [62];
- As knowledge and research increases, technology such as artificial intelligence can aid in faster decision-making and tracking of COVID-19 cases. It can be used in various applications, including:
  - Developing advanced diagnostic tests and vaccines;
  - Predicting vulnerable regions, people and countries in which measurements should be taken rapidly;
  - Providing data on the number of resources needed in certain hospitals, such as number of beds and ventilators [63].

**Figure 6.**
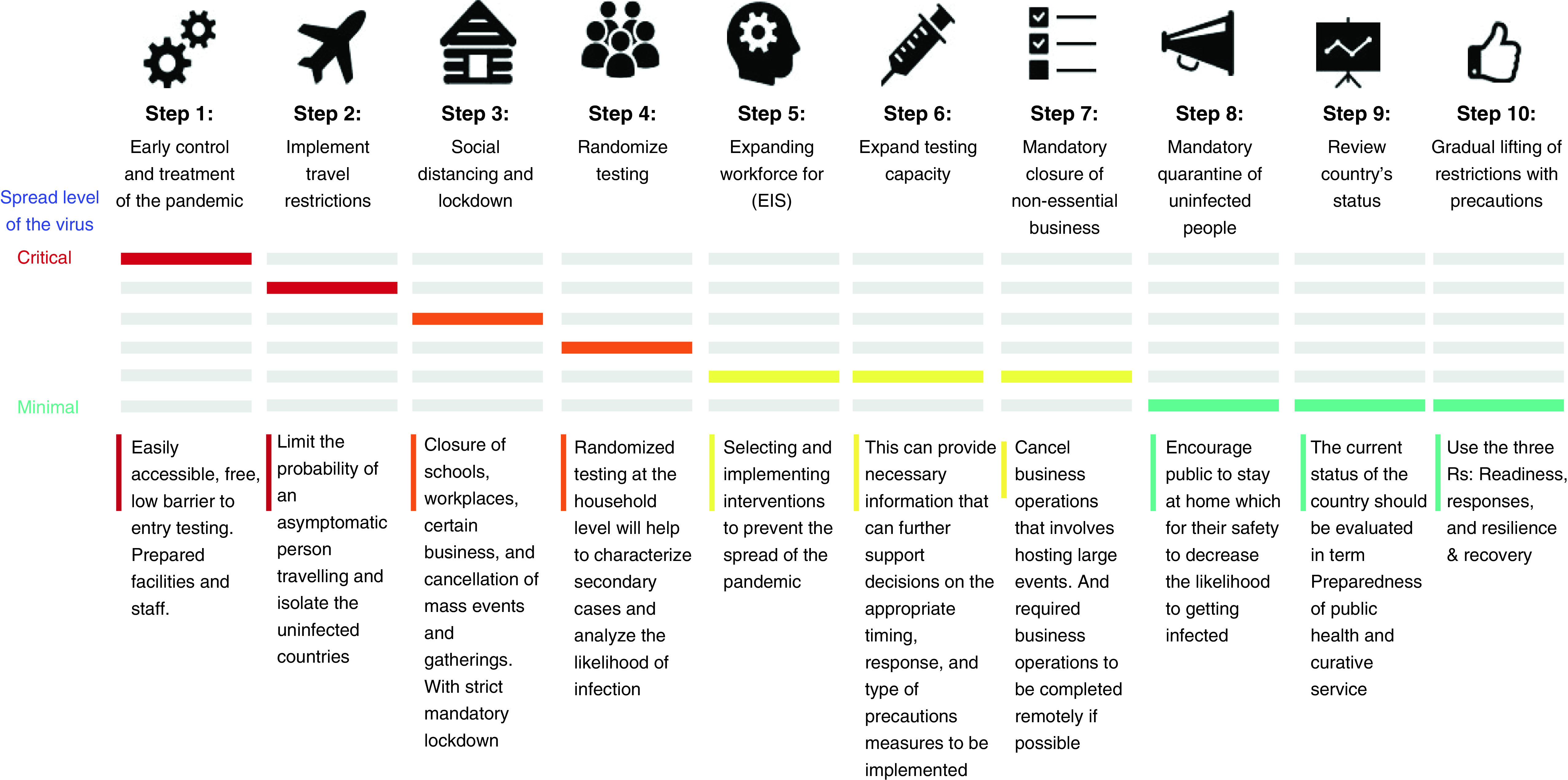
Action plan for decision-makers.

The lack of a global public health database support system compounded the complication and inefficiency of developing a robust and uniform response to COVID-19. Global collaboration and high-quality data sharing are needed to fight COVID-19 [64] and any similar pandemic. It is recommended that a global public health pandemic database monitoring and support system be established and supported by all countries, because a pandemic knows no border. Figure 6 summarizes an action plan for decision-makers based on the framework and considering the level of criticality of a pandemic. The action plan includes:

- Early control: the first initiative is to identify that a pandemic has started. The main goal at this stage is to minimize the spread;
- Implementation of travel restrictions: one of the early measures for controlling the pandemic is to restrict travel. This step is necessary to isolate the uninfected regions, as well as limiting the probability of an asymptomatic person traveling. In addition, other countries will benefit from travel restrictions that slow the global spread of the pandemic, especially at a stage where it is not contained at its sources [65];
- Implementation of social distancing/mandatory lockdown: social distancing or mandatory lockdown aims at reducing community spread of the pandemic. In terms of effectiveness, mandatory lockdown is a strict measure that restricts people from leaving their homes, apart from through necessity and at certain determined times. In addition, mandatory lockdown enables drastic reductions in social contact [65];
- Randomized testing: randomized testing at the population-wide level will help understanding of the country’s epidemiological status and of transmission within the population setting, as well as estimation of secondary attack rates. Randomized testing within random households will help to characterize secondary cases, analyze the range of clinical presentations and the expected likelihood of infection, and expose asymptomatic infections [66];
- Expansion of the Epidemic Intelligence Service (EIS) workforce: one of the ways a country can measure and control the spread of the pandemic is through the use of EIS technology. The main goal of EIS is to rapidly provide guidance when selecting and implementing interventions to prevent the spread of the pandemic when it arises;
- Expansion of testing capacity: another step is to ensure that testing capacities can be expanded in infected regions. This can provide necessary information to further support decisions on the appropriate timing, response and type of precautionary measures to be implemented [67];
- Mandatory closure of nonessential businesses: this step includes the closure of nonessential businesses to the public as well as nonessential on-site business operations;
- Mandatory quarantine of uninfected people: it is essential to encourage the public to limit unnecessary contact because the safety measures will not help in identifying asymptomatic individuals. It inhibits asymptomatic individuals from further infecting others, which subsequently impacts the testing policies and strains the healthcare system due to limited capacity [68];
- Review of the country's status: before lifting restrictions, the country's situation and performance should be evaluated in terms of resources (medical supply, healthcare staff and number of tests). The number of infected cases and population should be taken into account. Safety measures should be established, and strict mandatory regulation should be applied to maintain the results gained from the previous stages. Lessons learned from the rapid action will be considered in taking subsequent steps. The spread of the virus, the preparedness of public health and curative services to contain all new cases, the ability to minimize the risk of resurgence, and population awareness are other factors to consider [69].
- Gradual lifting of restrictions with precautions: the three Rs – readiness, responses and resilience/recovery – represent the systematic approach for lifting lockdowns taken in times of crisis. Readiness consists of coordination of emergency task forces, training and skills building capacity, and increasing preparedness for health resources and services. Responses include legislation and laws for managing the reopening at the provincial level; public engagement and involvement of stakeholders, public awareness and education through effective communication, and activating the role of the judicial police are essential factors for responsiveness. The last step, resilience and recovery, involves taking advantage of the existing database by documenting the lessons learned. It includes health resilience and surveillance assessments and public policy and priority-setting based on setting criteria for lifting the lockdown, beginning with vital public sectors such as health and food security and followed by other sectors in a gradual approach that provides enough time to control the virus after reopening and detecting any new or suspected cases and their contacts [69].

## Conclusion

COVID-19 has made a significant impact on human life. The particular response strategy implemented has an enormous impact on the outcome for the country. In this study, DEA models were used to estimate the efficiency of contagion control for 58 countries and treatment efficiency of 57 countries affected by the COVID-19 pandemic. The results show significant inefficiency in contagion control, hence the large number of confirmed cases and consequent rise in related deaths. 89.6% of countries were inefficient in the first phase; this figure increases in the second phase to reach 96.5%. Sensitivity analyses underline the importance of resources in fighting the pandemic, thus resource augmentation for strategic purposes is recommended.

Further examination of efficient countries shows that mask wearing, social distancing, quick isolation and testing are key practices for an efficient response. Furthermore, the results of the study are consistent with observational studies such as that of Khorram-Manesh *et al*. [70] that emphasize continuous assessment, communication and complete physical distancing among the initial key strategies. The proposed pandemic response management framework minimizes the potential for overwhelming spread of the virus and the chances of viral resurgence. The recommended action plan helps decision-makers to implement the framework at different levels of criticality. It is evident that collective and spontaneous measures across countries will also minimize the impact of the pandemic. Therefore establishment of a global public health pandemic monitoring and support system will help to organize a global effort toward defeating possible future pandemics.

The study has some limitations. The authors acknowledge the absence of data on the number of COVID-19 tests during the evaluated period; the absence/inconsistency in data on COVID-19 testing and the possibility of repetition within the dataset hindered the use of this indicator as an input variable. However, this limitation does not affect the credibility of the analysis, because further examination identified countries with reliable data on testing to have adequate testing capacity. However, a micro-analysis at national level should consider testing as an input after rigorous statistical checks.

## Future perspective

Integration of innovative technology in the early stages of the pandemic was limited. Future studies should analyze strategic utilization of innovative technologies such as artificial intelligence (AI)/machine learning in the response system. In addition, future studies can support the proposed framework by integrating AI/machine learning at stages that require tracking, predicting, and proper screening process. It could also account for the statistical limitation of repetitive data in indicators such as number of testing or unreported cases.

**Executive summary**

- The study involves a comprehensive relative efficiency analysis of COVID-19 response management systems based on contagion control and treatment in 58 countries.
- It includes a comprehensive review of the COVID-19 response management strategies of efficient and inefficient countries.
- A robust pandemic response management framework is developed to address the shortfall of existing pandemic response management systems.
- Action plans are proposed with a recommendation for a global public health pandemic database monitoring and support system as the nucleus.
