# Supplementary material for: Pandemic response management framework based on efficiency of COVID-19 control and treatment
Source: Future Virol. 2020 Dec 16:10.2217/fvl-2020-0368. doi: 10.2217/fvl-2020-0368 (PMC7740003; doi:10.2217/fvl-2020-0368)
Supplement: Supplementary file 1 [file fvl-2020-0368_supp_data.docx]

**Supplementary Tables & Figures**

Supplementary Table 1: Descriptive statistics

| Stage 1: Efficiency of COVID-19 contagion control | | | | | | |
| --- | --- | --- | --- | --- | --- | --- |
|  | Popltn.  Density | Avg. of 13 IHR | Confirmed cases (Stage1A) | Confirmed cases (Stage1B) |  |  |
| Mean | 338.64044 | 76.06896552 | 12415.966 | 209770.31 |  |  |
| Std. Dev. | 1122.5531 | 16.22307439 | 27995.222 | 513501.15 |  |  |
| Min. | 3.3193029 | 34 | 5 | 2701 |  |  |
| Max. | 8357.6329 | 99 | 140640 | 3358651 |  |  |
| Stage 2: Efficiency of COVID-19 treatment | | | | | | |
|  | Confirmed  cases | No. of Physicians  per 1000 popltn. | No. of Hospital beds  per 1000 popltn. | % of population  with age above 65 | COVID-19  related deaths | COVID-19  recovered cases |
| Mean | 224742.32 | 2.264050877 | 3.2877193 | 10.813381 | 125874.81 | 9826.193 |
| Std. Dev. | 532135.27 | 1.345684492 | 2.8398359 | 6.9460774 | 241823.67 | 21983.485 |
| Min. | 10810 | 0.1359 | 0.3 | 1.1565495 | 193 | 27 |
| Max. | 3499291 | 5.1697 | 13.4 | 28.002049 | 1350098 | 137419 |


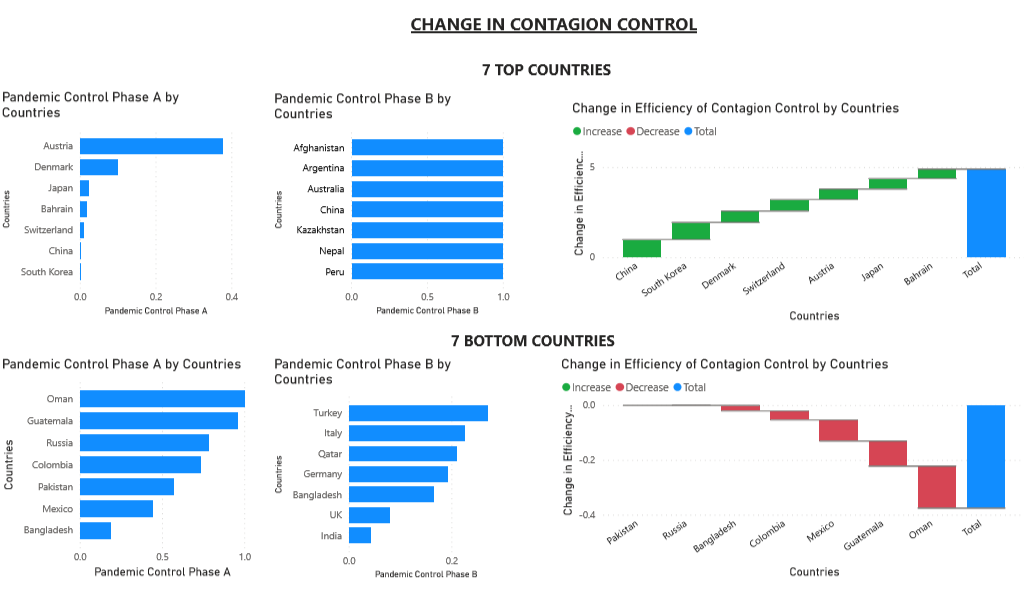


Supplementary Figure 1: Change in Efficiency of Contagion control

Supplementary Table 2: Efficiency of COVID-19 Contagion control

| Countries | Stage1A | Stage1B | Efficiency Difference |
| --- | --- | --- | --- |
| Afghanistan | 100.0% | 100.0% | 0.0% |
| Algeria | 87.6% | 97.9% | 10.3% |
| Argentina | 100.0% | 100.0% | 0.0% |
| Australia | 100.0% | 100.0% | 0.0% |
| Austria | 37.7% | 96.7% | 59.0% |
| Azerbaijan | 53.8% | 66.8% | 13.0% |
| Bahrain | 1.7% | 54.1% | 52.3% |
| Bangladesh | 18.5% | 16.6% | -2.0% |
| Belgium | 0.2% | 36.1% | 35.9% |
| Brazil | 54.2% | 54.2% | 0.0% |
| Canada | 90.8% | 90.8% | 0.0% |
| Chile | 66.8% | 66.8% | 0.0% |
| China | 0.3% | 100.0% | 99.7% |
| Colombia | 73.3% | 70.0% | -3.3% |
| Denmark | 10.0% | 74.0% | 64.0% |
| Dominican Republic | 52.2% | 64.4% | 12.2% |
| Egypt | 36.3% | 47.9% | 11.6% |
| France | 8.6% | 34.8% | 26.3% |
| Germany | 0.3% | 19.2% | 19.0% |
| Ghana | 80.3% | 95.7% | 15.3% |
| Guatemala | 96.0% | 86.8% | -9.2% |
| Honduras | 90.4% | 94.7% | 4.3% |
| India | 0.8% | 4.2% | 3.4% |
| Indonesia | 23.6% | 52.1% | 28.6% |
| Iran | 69.5% | 75.0% | 5.6% |
| Iraq | 63.5% | 73.0% | 9.5% |
| Ireland | 56.0% | 90.0% | 34.0% |
| Italy | 0.2% | 22.6% | 22.3% |
| Japan | 2.4% | 60.2% | 57.8% |
| Kazakhstan | 100.0% | 100.0% | 0.0% |
| Kuwait | 32.6% | 41.1% | 8.5% |
| Mexico | 44.2% | 36.5% | -7.7% |
| Morocco | 45.4% | 89.4% | 43.9% |
| Nepal | 100.0% | 100.0% | 0.0% |
| Netherland | 0.1% | 39.0% | 38.9% |
| Nigeria | 61.4% | 71.0% | 9.6% |
| Oman | 100.0% | 84.7% | -15.3% |
| Pakistan | 56.7% | 56.7% | 0.0% |
| Panama | 54.8% | 76.5% | 21.7% |
| Peru | 100.0% | 100.0% | 0.0% |
| Philippines | 44.1% | 53.8% | 9.7% |
| Poland | 33.7% | 69.2% | 35.6% |
| Portugal | 10.0% | 60.6% | 50.7% |
| Qatar | 14.6% | 20.9% | 6.4% |
| Romania | 46.8% | 83.7% | 36.9% |
| Russia | 78.1% | 78.1% | 0.0% |
| Saudi Arabia | 86.3% | 86.3% | 0.0% |
| Singapore | 1.1% | 35.7% | 34.6% |
| South Africa | 60.0% | 65.0% | 5.0% |
| South Korea | 0.1% | 95.3% | 95.2% |
| Spain | 9.2% | 28.2% | 19.0% |
| Sweden | 49.8% | 68.3% | 18.5% |
| Switzerland | 1.1% | 64.2% | 63.1% |
| Turkey | 20.6% | 27.0% | 6.4% |
| UAE | 31.3% | 44.7% | 13.3% |
| UK | 0.5% | 8.0% | 7.5% |
| Ukraine | 57.9% | 72.0% | 14.1% |
| USA | 30.4% | 30.4% | 0.0% |

Supplementary Table 3: Efficiency of COVID-19 Treatment

| Country | Model 1 | Model 2 |
| --- | --- | --- |
| Afghanistan | 60% | 49% |
| Algeria | 64% | 64% |
| Argentina | 5% | 3% |
| Australia | 70% | 70% |
| Austria | 92% | 92% |
| Azerbaijan | 51% | 51% |
| Bahrain | 90% | 90% |
| Bangladesh | 100% | 22% |
| Belgium | 0% | 0% |
| Brazil | 100% | 59% |
| Canada | 55% | 55% |
| Chile | 100% | 94% |
| China | 97% | 97% |
| Colombia | 18% | 1% |
| Denmark | 96% | 96% |
| Dominican Republic | 6% | 6% |
| Egypt | 10% | 1% |
| France | 1% | 0% |
| Germany | 96% | 95% |
| Ghana | 100% | 86% |
| Guatemala | 1% | 0% |
| Honduras | 1% | 1% |
| India | 100% | 47% |
| Indonesia | 35% | 3% |
| Iran | 100% | 87% |
| Iraq | 55% | 46% |
| Ireland | 94% | 94% |
| Italy | 83% | 80% |
| Japan | 76% | 76% |
| Kazakhstan | 38% | 38% |
| Kuwait | 83% | 83% |
| Mexico | 96% | 78% |
| Morocco | 86% | 86% |
| Nepal | 55% | 52% |
| Netherland | 0% | 0% |
| Nigeria | 23% | 2% |
| Oman | 48% | 48% |
| Pakistan | 100% | 60% |
| Panama | 12% | 12% |
| Peru | 84% | 56% |
| Philippines | 12% | 2% |
| Poland | 68% | 68% |
| Portugal | 57% | 57% |
| Qatar | 100% | 100% |
| Romania | 50% | 50% |
| Russia | 100% | 62% |
| Saudi Arabia | 100% | 73% |
| Singapore | 100% | 100% |
| South Africa | 98% | 13% |
| South Korea | 94% | 94% |
| Spain | 39% | 34% |
| Switzerland | 92% | 92% |
| Turkey | 100% | 94% |
| UAE | 84% | 84% |
| UK | 0% | 0% |
| Ukraine | 10% | 10% |
| USA | 50% | 1% |
